# Synthetic polymeric biomaterials for wound healing: a review

**DOI:** 10.1007/s40204-018-0083-4

**Published:** 2018-02-14

**Authors:** Mariam Mir, Murtaza Najabat Ali, Afifa Barakullah, Ayesha Gulzar, Munam Arshad, Shizza Fatima, Maliha Asad

**Affiliations:** Biomedical Engineering and Sciences Department, School of Mechanical and Manufacturing Engineering (SMME), National University of Sciences and Technology (NUST), Sector H-12, Islamabad, Pakistan

**Keywords:** Polymeric biomaterials, Wound healing, Bio-engineering skin substitutes, Hydrogels, Hydrocolloids

## Abstract

Wounds are of a variety of types and each category has its own distinctive healing requirements. This realization has spurred the development of a myriad of wound dressings, each with specific characteristics. It is unrealistic to expect a singular dressing to embrace all characteristics that would fulfill generic needs for wound healing. However, each dressing may approach the ideal requirements by deviating from the ‘one size fits all approach’, if it conforms strictly to the specifications of the wound and the patient. Indeed, a functional wound dressing should achieve healing of the wound with minimal time and cost expenditures. This article offers an insight into several different types of polymeric materials clinically used in wound dressings and the events taking place at cellular level, which aid the process of healing, while the biomaterial dressing interacts with the body tissue. Hence, the significance of using synthetic polymer films, foam dressings, hydrocolloids, alginate dressings, and hydrogels has been reviewed, and the properties of these materials that conform to wound-healing requirements have been explored. A special section on bioactive dressings and bioengineered skin substitutes that play an active part in healing process has been re-examined in this work.

## Introduction

For effective wound healing to occur, there has always existed a requirement for a suitable material that would cover the wound to prevent infection (Majno 2014). Biopolymers, materials such as animal fats, plant fibers, and honey pastes, were commonly used in the past as wound dressings (Thomas^1990^). A few years later, materials like cotton wool, lint, and gauzes started being used as dressings, and their main function was to keep the wound dry by allowing wound exudates to evaporate and inhibiting invasion of bacteria from the surrounding environment (Thomas 1990). Recent studies have proven that a moist and warm environment provides a better alternative to the previous wound-healing therapies. The compatibility of the physical and chemical properties of the dressing towards the nature of the wound must take into account the designing a bandage for wound healing. An active wound dressing controls the biochemical state of the wound to aid the healing process. No wound dressing is ideal, but the minimum requirements of rapid healing, affordable cost to the patient, aesthetics, and prevention of infection, must be fulfilled during wound management (Helfman et al. 1994).

Wounds are an unnatural break, tear, or defect in the skin, caused by thermal/physical injury, or an underlying pathological condition (Percival 2002). Based on the repair process, wounds may be classified as acute or chronic wounds. Acute wounds are tissue injuries mostly tend to heal completely, usually within a time frame of 8–12 weeks and with minimal scarring. Chronic wounds generally tend to reoccur and have a healing time extending beyond 12 weeks (Bryant and Nix 2006). Underlying physiological conditions may result in delayed healing of a wound, or complete failure of a wound to heal. Bed sores and leg ulcers (ischemic or venous) are examples of chronic wounds (Mostow 1994).

Skin layers and affected areas are often used as the basis for gradation of wounds. Superficial wounds are those that involve only the epidermal skin surface. Injuries that involve the epidermis, deeper dermal layers, blood vessels, sweat glands, and hair follicles are known as “partial thickness wound”. Full thickness wounds are caused when the subcutaneous fat or deeper tissue along with the epidermis and dermal layers is injured (Bolton and Van Rijswijk 1991).

The physiological process of wound healing involves coordinated interaction amongst various biological systems. It involves cascade of regulated events which rely on each other to completely close a wound (Hunt et al. 2000).

As a lesion appears in any organ of the body, hemostasis, and coagulation begins. The primary aim of these processes is to prevent immediate exsanguinations. However, a secondary, long term, aim is to provide invading cells with a matrix for attachment (Lawrence 1998). Homeostasis and amount of fibrin deposited at the wound site depend on a carefully regulated balance between endothelial cells, thrombocytes, coagulation, and fibrinolysis. Vasoconstriction takes place in injured vessels due to neural reflex mechanism, thereby ceasing blood flow occurs for a few minutes. This does not last for a long period of time. As hypoxia and acidosis build up at the affected site, passive relaxation takes place and bleeding resumes. This is where insoluble fibrin plug plays an important part, were it for homeostasis alone, the mechanism would be ineffective for long duration. Homeostatic events, together with intrinsic and extrinsic pathways, activate coagulation cascade, leading to clot formation and limiting blood flow. As blood spills, platelets attach themselves to extracellular matrix, thus triggering the release of clotting factor: fibronectin, fibrin, vitronectin, and thrombospondin. Blood clot not only maintains homeostasis but, in subsequent homeostatic and inflammatory phase, also provides the migrating cells with a matrix (Strecker-mcgraw et al. 2007).

Inflammatory phase proceeds coagulation and homeostasis for developing an immune barrier against foreign invasion. Inflammatory phase appears in the early and late phases.

Early inflammatory response is initiated in the late phase of coagulation. Chemo-attractive agents, TGF-β, complement components such as C3a and C5a, and formyl–methionyl peptides, produced by bacteria and platelets, are responsible for bringing neutrophils to the site of injury. This process occurs with 24–36 h of injury (Hart 2002). Alteration in adhesion molecules renders neutrophils sticky, and by the process of margination, they adhere to post-capillary venules, rolling along the surface of endothelium as blood flows. Adhesion and rolling are classified as weak attachments mediated by selecting dependent interactions. Strong adhesion system, mediated by integrin, is activated when endothelial cells release chemokines. Following strong adhesion, cell rolling and attachment come to a halt and are sequestered from post-capillary venule. Neutrophils now reside between endothelial cells. This process is known as diapedesis (Richardson 2004). Once in the wound milieu, neutrophil releases proteolytic enzymes and free oxygen radicals, thereby killing and phagocytosing the bacteria. After completing their task, neutrophils must be eliminated if wound healing is to proceed further. They are disposed as slough from wound surface by apoptosis. Apoptotic bodies are phagocytosed by macrophages without invoking an inflammatory response (Diegelmann and Evans 2004).

As for late inflammatory phase, macrophage migration continues by chemokines. They have a longer life span and can work in low pH environment. These cells are a reservoir for growth factor (TGFβ), mediators (TGFα and FGF) and activating keratinocytes, fibroblasts, and endothelial cells. Lymphocytes, attracted by interleukin-1, complement component, and immunoglobulin G, are the last cells to enter inflammatory phase. IL-1 has an important part to play in collagen regulation, remodeling, and extracellular matrix formation (Hunt 1988).

Proceeding first two phases of wound healing is a repairing phase, proliferative phase. It begins 3 days after the injury and lasts for 2 weeks. After injury, fibroblast and myofibroblast proliferate in local wound milieu and are stimulated by TGFβ and PDGF, to migrate to the site of injury on the third day, where they proliferate profusely. Fibroblast lays extracellular matrix of matrix proteins: hyaluronan, fibronectin, proteoglycans, and type 1 and type 3 procollagen. This matrix helps support cell migration and repair process. Wound contraction, an important reparative process, to approximate wound edges takes place after which fibroblasts are eliminated (Hunt 1988).

Angiogenesis takes place when endothelial cells are stimulated by angiogenic factors, i.e., FGF, vascular endothelial growth factor (VEGF), PDGF, angiogenin, TGFα, and TGFβ. This process is kept in check and regulated by interaction of angiogenic factors and angiostatin (Oike et al. 2004). Under hypoxic conditions, endothelial cells release proteases to degrade basal lamina followed by chemotaxis, proliferation, and remodeling. Initially, vascular supply is limited to wound margins; then, capillary sprouts and a microvascular network appears (Phillips 2000).

Remodeling is the last phase of wound healing. It is tightly regulated by degradation and synthesis, leading to normal wound healing (Baum and Arpey 2005). Matrix metalloproteinases, produced by neutrophils, macrophages, and fibroblasts in the wound, are responsible for breakdown of collagen. This breakdown is synchronized with activity of inhibitory factors. Initial deposition of collagen is disorganized; however, it becomes organized over a period of time (Clark 1993). The underlying connective tissue shrinks and brings wound margins close together. Growth of capillaries ceases, blood flow, and thus, the metabolic activity declines leading to formation of matured scar with few cells and high tensile strength (Han and Ceilley 2017).

## Factors affecting wound healing

Numerous factors which influence wound healing can impair the whole process. In general, these factors are classified as local and systemic (Guo and Dipietro 2010). Local factors, oxygenation and infection, directly affect wound healing. Oxygen is critical for cell metabolism, energy production, and for all phases of wound healing. Superoxide, for oxidative killing of pathogens, produced by leukocytes is dependent on oxygen levels. In wounded region, vascular disruption causes depletion of oxygen leading to hypoxia. In wounds, where oxygenation is not restored, healing is impaired. Temporary hypoxia stimulates wound healing, while chronic hypoxia delays the process. Hypoxia induces macrophages, fibroblasts, and keratinocytes to produce cytokines and growth factors crucial for cell proliferation migration and chemotaxis, and angiogenesis in wound healing. Reactive oxygen species, during normal oxygenation, induce wound healing. However, in case of hypoxia, as their levels increase in cell, their beneficial effects are thwarted by their harmful effects of tissue damage (Bishop 2008).

Once skin is injured, the barrier it provides against foreign invasion is breached. Instead of being sequestered at skin surface, microorganisms enter the wound and contaminate or colonize it. Inflammation is an important part of wound healing. However, improper removal of bacteria from wound leads to prolonged elevation of pro-inflammatory cytokines and matrix metalloproteinases. Enhanced production of proteases can recede the production of naturally occurring proteases. Imbalanced production of protease degrades growth factors allowing bacteria to form a biofilm. Infected wounds are teeming with *Staphylococcus aureus* (*S. aureus*), *Pseudomonas aeruginosa* (*P. aeruginosa*), and β-hemolytic *streptococci* (Guo and Dipietro 2010).

Systemic factors are essential in determining the overall well-being of an individual on wound healing. Increased age is a risk factor for impaired wound healing. It hampers the process by altering inflammatory responses, re-epithelialization, collagen synthesis, and angiogenesis in aged mice as compared to young ones. Female estrogen and male androgens play an important role in wound healing. Estrogen is known to regulate genes involved in regeneration, matrix production, protease inhibition, epidermal functions, and inflammation (Guo and Dipietro 2010).

Stress causes substantial delay in wound healing. Glucocorticoids are up-regulated in stress, reducing the levels of pro-inflammatory cytokines and chemo-attractants, necessary for inflammatory phase of wound healing. Glucocorticoids induce reduced differentiation and proliferation of immune cells, cell adhesion molecules, and expression of gene-regulating transcription (Godbout and Glaser 2006).

Diabetic individuals show delayed and impaired wound healing. Such individuals are prone to diabetic foot ulcer (DFU) which subsequently causes lower limb amputation in 80% of cases. DFU is accompanied by hypoxia which causes insufficient angiogenesis and amplifies early inflammatory response and enhanced levels of oxygen radicals. Hyperglycemia boosts ROS level, adding oxidative stress. High levels of MMP in DFU, 60 times higher than acute wounds, distort tissues, and impede wound healing (Brem and Tomic-Canic 2007).

Neuropeptides, important for cell chemotaxis, growth, and proliferation, recede substantially, thus affecting the process of wound healing. Obesity, being a leading risk factor for myriad diseases and health conditions, affects wound-healing process. High infection rates are reported in obese individuals undergoing bariatric and non-bariatric surgeries. Hypo-vascularity in wounded area also contributes towards impaired healing. Adipose tissues and macrophages within contain bioactive adipokines. Negative influence of adipokines impairs wound-healing process (Wilson and Clark 2004).

Clinical evidence and experimental studies suggest alcohol consumption as a risk factor for impaired wound healing. Exposure to alcohol interferes with defense mechanism rendering wound vulnerable to further infection. Most significant impairment is due to reduced angiogenesis (Radek et al. 2005). Like alcohol consumption, smoking has deleterious effects on wound healing. Compounds of cigarette smoke interfere with the process. Nicotine causes vasoconstriction and reduces blood perfusion. Carbon monoxide compromises oxygen consumption. Despite overall negative outcomes, recent studies put forth low dose of nicotine to stimulate angiogenesis (Ahn et al. 2008).

## Types of dressings and polymers used for wound healing

It is a widely accepted hypothesis that moist wound dressings promote a faster rate of wound healing as compared to dry wound dressings. This hypothesis was put to test through winter’s experiments who showed that epithelialization occurs twice as fast in young domestic pigs with moist wound dressings as compared to those with a dry wound dressing. Scab formation was also considered preventive in moist wound conditions (Winter 1962) (Table 1).

**Table 1:** Commercially available wound dressings (195)

Type of polymeric dressing	Brand name^®^	Use for
Polymeric foam	Flexzan	Chronic wounds
	Biopatch	
	Crafoams	Burns
	Biatain	Mohs surgery and wounds
	Cutinova	Laser resurfacing wounds
Polymeric hydrogels	Cultinova Gel	Chemotherapy peels
	Biolex	
	TegaGel	
	2nd skin Flexderm	Ulcers
	Dry dressing	Laser resurfacing
Polymeric alginates	AlgiSite	Thickness burns
	AlginSan	
	Sorbsan	Surgical wounds
	Kaltostat	High exudate wounds
	Omiderm	Chronic ulcer
Polymeric hydrocolloides	Idosorb	Chronic ulcer
	Debrisan	Burns
	Sorbex	Average thickness wounds
	Duoderm	

Subsequent studies on dermal wound healing have taken into account the proliferating rates of cellular entities involved in wound healing. Comparing the effects of moist and dry wound conditions affecting the populations of both kinds of cells shows that the inflammatory phase cells (neutrophils and macrophages) fail to multiply rapidly in a moist wound environment. Whereas the numbers of endothelial and fibroblast cells increase in moist wound conditions as compared to wounds that are kept dry (Dyson et al. 1988).

Movement of epithelial cells across wound surfaces is facilitated in wounds that are kept moist and scab free. This in turn promotes wound healing (Rovee et al. 1972). Superficial wounds are susceptible to pronounced scar formation in some individuals. Wounds that are kept moist are prone to less obvious scab formation, thus alleviating cosmetic concerns (Atiyeh et al. 2003a, b). Reduction in pain symptoms of patients has been observed in moist wound-healing environments (Flanagan and Marks-Maran 1997).

Occlusive dressings that are now commonly used act by keeping the wound sealed, so that moisture is trapped in the wound. Thus, the wound is kept safe from desiccation and further damaging of the tissue from exposure to the environment. As explained earlier, desiccation and trauma hinder the migration of epithelial cells towards the damaged site (Rheinecker 1995). Since epithelial cells have a key role in wound healing, this parameter should be kept in consideration when designing a dressing to cover the wound.

Prevention of wound desiccation results in the formation of an electrical potential between the moist environment of the wounded tissue and the drier area of the non-wounded surrounding tissue. This stimulates the migration of epithelial cells towards the wound site. Expression of growth factors on fibroblast cells also increases after providing electrical stimuli. Both these factors aid wound healing (Mertz et al. 1993; Falanga et al. 1987). Occlusive dressings are costly but have been shown to give effective results in treatment of wounds with shorter nursing period (Helfman et al. 1994).

### Synthetic polymer films

Polymer films have been initially introduced as drapes for surgical incisions. Studies have documented the use of polymers in dressings or as medical devices for potential improvement in controlling wound healing (Mir et al. 2014a, b, 2016). However later they have now carved a niche in wound-healing management as occlusive dressings.

During the past years wound dressing has been modernized which now mainly consist of synthetic polymers for wound management. Synthetic polymer dressings can be classified as passive and interactive. Passive synthetic polymer dressings are non-occlusive, used for covering wound and help in restoring function under the polymer film. Example of such passive synthetic polymers is gauze and tulle. Interactive synthetic polymer dressings are occlusive or semi-occlusive which provide a barrier against bacterial penetration to the wound. They can be in the form of films, foams, hydrogel or hydrocolloids (Dhivya et al. 2015).

Polymer films trap exudates, hence providing a moist environment for wounds. Polyurethane (PU) is used in many semi-permeable dressings because of its ability to provide good barrier and permeability to oxygen. One main feature is that these films are impermeable to bacteria and liquid but are permeable to moisture vapor and air. Exudates from wounds may accumulate underneath such semi-occlusive film since these dressings are non-absorbent which is a major drawback of such dressings. Although this does not appear to encourage bacterial growth in the wound, the seeping fluid pressure may cause a break in the environment maintained by the occlusive dressing.

In one of the experiment, synthetic polyurethane films have been analyzed as potential polymeric wound dressings. As compared to the control dressing Tegaderm™ that is commercially in use for healing of wounds and it is considered as standard dressing (Jenks et al. 2016), wounds covered with polyurethane films form a thinner scab, with lower inflammatory cell infiltration. The study also shows earlier formation of granulation tissue on the wound site, which is vascularized and rich with collagen. Wound healing also depicts a better rate of epithelial cell organization as compared to Tegaderm™ which has been used as a the control (Khil et al. 2003).

## Foams

Another class of occlusive polymeric dressings includes foam dressings. Foams became widely available in the mid-1979s for exuding wounds and were one of the first ‘modern’ dressings to be used for wound dressings. Foams have wide advantages over traditional gauze dressings as they do not shed particles and can be left in place for several days without causing maceration. Physical and chemical properties of the wound dressings such as foam dressing play an important role in creating environment conductive to the wound healing (Zahedi et al. 2010).

Wet environment is important for the fastest wound healing. Polyurethane foam film dressing made of microporous upper layer and sponge like porous sublayer. It provides the effectiveness in the prevention of bacterial infection and dehydration (Hinrichs et al. 1992).

The aim of selecting a suitable dressing is to create an optimal environment that facilitates healing by providing protection from infection, contamination and enhancing the activity of enzymatic system to promote epithelialization and controlling the biomechanics of the wound area to provide mechanical stability. In other words, physical properties such as thickness, porosity, density and fluid absorption/retention capacity of wound-healing materials play an important role in creating optimal environment for wound healing. Thus, smaller pore size with excellent uniformity improves the fluid retention/absorption capacity of the dressing (Lee et al. 2016).

Foam dressings attempt to rectify the lack of exudate absorbancy of occlusive dressing, without compromising on the moist environment needed for tissue repair (Helfman et al. 1994). The effect of a hydrophilic polymeric foam dressing was observed on 12 patients suffering from venous leg ulcers in a single blinded cross over study. An analgesic agent, which was released in the presence of wound exudates was also an integral part of the matrix. A semi-permeable polyurethane film was bonded to the matrix. The use of polyurethane has been reported in many biomedical applications (Munib et al. 2015). Favorable results were seen regarding “quality of life” (QOL) assessments and pain reduction (Jørgensen et al. 2006).This is a significant finding since wound pain may potentiate a more aggravated sympathetic nervous system response and hence an increase in the release of catecholamines. This leads to peripheral arteriolar constriction and a decrease in oxygen supply to the site of the wound, which in turn has the effect of impeding the wound-healing process and increasing susceptibility to infections (Jørgensen et al. 2005). Silver releasing foam dressings have been tested in open randomized control groups where the effect of these dressings on ulcer area and healing were observed. The results showed less maceration and wound leakages in patients treated with silver releasing dressings when compared with those without silver, outlining this as a better option for chronic wound treatment (Burey et al. 2008).

## Hydrocolloids

Hydrocolloid dressings are occlusive, absorbent and semi-permeable to vapor. Hydrocolloids are polymers with hydrophilic properties due to the presence of many hydroxyl groups. Hydrocolloids can be obtained synthetically or naturally. The type of hydrocolloids used in practical applications is generally polysaccharides (Jeffcoate et al. 2004).

Since hydrocolloid dressings are occlusive, they maintain a hypoxic environment which leads to liquefaction of necrotic tissue and aids autolytic debridement. Case studies reporting ease of autolytic debridement of diabetic foot ulcers following treatment with hydrocolloid dressings have strengthened this hypothesis (Hutchinson and Lawrence 1991). There is some evidence that hydrocolloids increase leukocyte infiltration and thus decrease risk of infection by preventing the spread of MRSA (methicillin resistant *Staphylococcus aureus*) strains (Nguyen et al. 2013).

Formation of granulation tissue on exposed bone wound (without periosteum) is usually a reconstructive surgical challenge since the wound bed is poorly vascularized. However, healthy granulation layer formation has been observed after application of hydrocolloid dressing to the wound (Das and Baker 2016).

In medical applications, the current market for hydrocolloid dressings makes its uses in healing diabetic foot ulcers.

Hydrocolloids vary in origins based on the industrial needs. Some of the types of hydrocolloid gels are as following examples (Das and Baker 2016).

### Agar

Agarose, commonly known as agar is one type of hydrocolloid gel, which is obtained from the special type of seaweeds namely, gelidium and gracilaria. The gelling of agar occurs when formation of the double helices in the structure occurs, which is known as cold-set mechanism. The use of agar with other gums like xanthan is known to yield materials with improved characteristics (Mir et al. 2014a, b).

### Alginate

Another type of hydrocolloid gel which is obtained from a seaweed (Phaeophyceae). These types of hydrocolloids are linear and unbranched. The gel forming in alginates involves the ionotropic mechanism which is enhanced when there are divalent cations present during the gelling of the hydrocolloid.

### Carrageenan

The hydrocolloid, extracted from the sea weed class Rhodophyceae, are known as carrageenans. Carrageenans are made up of multiple interlinked structures as compared to alginate structure. Carrageenans are commercially available as a mixture of three types of carrageenan.

The gel setting of carrageenan involves both the ionotropic and cold-set mechanism.

### Pectin

Pectin is a hydrocolloid which is rather found in the land based plants instead of seaweeds. The linear chains of galacturonic acid make up the basic chemical structure of pectin. The gelation of pectin hydrocolloids is dependent upon the degree of esterification of that specific pectin type. Low ester pectins form gel structure using the ionotropic mechanism as compared to the gelation of high ester pectins which gel when provided with a low amount of pH and high soluble solids.

### Gelatin

Unlike the rest of hydrocolloids which are obtained from plants, gelatin is a special type of hydrocolloid which is obtained from an animal protein named collagen. Gelatin based hydrocolloids are thermoreversible hydrocolloids which form gels based on cold-setting mechanism. Gelatin requires an aqueous solvent for the cold-setting gelation.

The use of hydrocolloid gels range from texturing and structuring of an application to the encapsulation of material in the hydrocolloid gel. In medical applications and specifically in wound-healing applications, the encapsulation property of the hydrocolloid has the highest use (Das and Baker 2016). The encapsulation properties of hydrocolloids provide an opportunity to create such solutions where the requirement is of slow release or bulk release of drugs or any other product in the surrounding system. The different type of hydrocolloid gels with different structural gelation provides the hydrocolloid dressings with an opportunity to exhibit the control of drug release. The amount of drug release can be controlled by optimization of the size of particles and the permeability of the gel membrane (Bramhill et al. 2017).

Hydrocolloid dressings contain gel forming agents that are mounted on flexible and water resistant layers. Some contain alginates to enhance water absorption capacity. Such dressings are self-adhesive and can have various thicknesses and cut in shapes according to the shape of body part. Carboxymethylcellulose is the most common absorbent agent in hydrocolloid dressings. Obsolete versions of hydrocolloid were completely occlusive. However, nowadays semi-occlusive versions are also available which do not allow passage of fluid and bacteria but are semi-permeable to gas and water vapors. Relatively low oxygen tension stimulates angiogenesis and fibroblast and epidermal cell turnover and, therefore, it is expected to provide good conditions for wound healing. Examples of commercially available products include Duoderm (Convatec), Granuflex (Convatec), and Comfeel (Coloplast).

The adhesive side of dressing adheres to wet and dry skin but does not stick to the wound bed. Being waterproof, hydrocolloid dressings provide comfortability and flexibility. Dressing absorbs fluid from the wound and hydrocolloid forms viscous gel that provides moist environment with reduces local pH. Upon removal of hydrocolloid, wound fluid mixes with gel and produces pungent odor that might be mistaken for an infection. Contoured dressings are available suitable for anatomically challenging sites e.g., heel, knee and elbow. Adhesive borders of hydrocolloid dressings extend beyond the gel so to circumvent the need for additional adhering substances. Hydrocolloid dressings are designed on some wounds for prolonged periods (more than 1 week); this is useful in managing clean ulcers, but not when regular wound inspection is required. Thus, these dressings are probably more useful in preventing, rather than treating, infection within a wound.

**Fig. 1:**
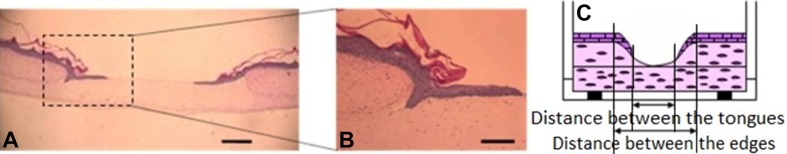
Eosin and hematoxylin staining shown. In **a**, **b,** the epithelial tissue migrates across the surface of the wound to close the wound. In **c**, the distance between wound edges decreases slowly until the wound closes (Schneider et al. 2008)

Management of chronic wounds is a common problem in health care. Hydrocolloid s have been promoted as an effective method for healing chronic wounds. Immediate benefits include a moist, warm, hypoxic and contamination-free environment that promotes wound healing. Moreover, patients require fewer outpatient visits and mobility increases markedly as the dressing remains in situ for 7–10 days and is replaced only when it leaks. Since hydrocolloid is semi-permeable, patients can take regular baths or even swim without the need for a dressing change. Multiple meta-analysis put forth that hydrocolloid dressings are much better for wounds than gauze. Healing rates of these types of dressings are high, similar to other moist healing dressings (Frykberg and Banks 2015) (Fig. 1).

## Hydrogels

**Fig. 2:**
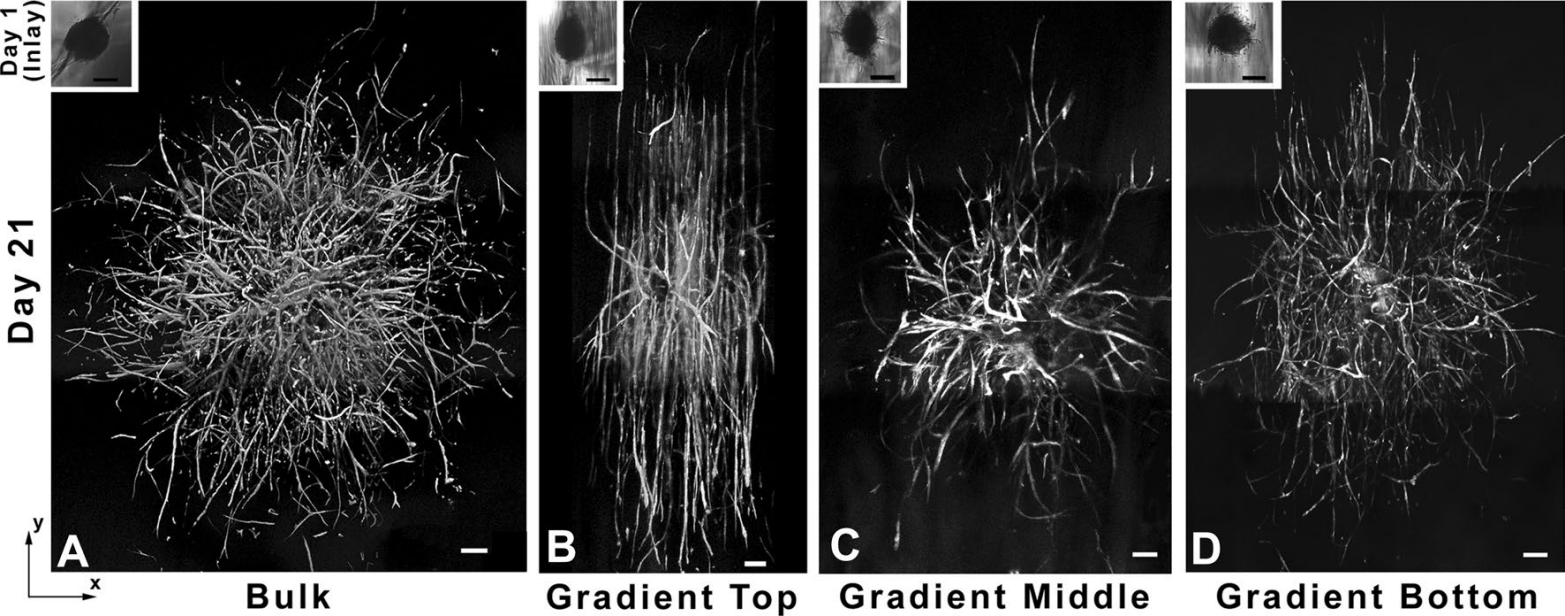
Three-dimensional mosaic rendition of cell aggregates that have been seeded in **a** control hydrogels, **b** top, **c** middle, and **d** bottom portions of PBFP–polyethylene (glycol) diacrylate gradient hydrogels. The images show vascular sprout in the gradient hydrogel regions. Scalebar 200 µm (Turturro et al. 2013)

Hydrogels are made of crosslinked polymers (hydrophilic) such as polyvinylpyrrolidone, polyacrylamide, and polyethylene oxide. Hydrogels do not dissolve in water and have the innate characteristics of swelling when in contact with water (Najabat Ali et al. 2016). Most hydrogels do not adhere naturally to skin and so require a secondary layer to fasten them in position. Hydrogels are employed as wound dressings in the shape of elastic films or amorphous gels (3, 60). Vascular sprout has been observed in hydrogels (see Fig. 2).

The efficacy of drug free hydrogels for pain treatment of wounds in denture patients has been evaluated. This cross over study was conducted on 44 patients, and results indicated a statistically significant rate of pain relief in patients who had been treated with the hydrogel in addition to narcotics, as compared to patients who were treated with the narcotics alone (Kennedy and Hall 2008).

In vitro antimicrobial studies have been conducted to compare three types of burn dressings, a novel silver hydrogel dressing, a polymer film dressing and a polymer foam dressing. The antimicrobial activity of the silver hydrogel dressing has been shown to be superior to the other two dressings, despite comparative silver content in all three (Boonkaew et al. 2013). It has been found that the release of macromolecules from a hydrogel may be controlled by its hydrophobicity (Martin et al. 2002). However, most hydrogels do not have good mechanical properties and this might have a negative impact on patient compliance (Stammen et al. 2001).

In Fig. 2, F-actin staining was used for the visualization of sprout invasion after 3 weeks in culture. In Fig. 2a, sprout invasion was uniform and random in all directions. In Fig. 2b, sprout invasion was curved and invaded bi-directionally up and down the gradient. In Fig. 2c, d, aggregates seeded in the middle and bottom of hydrogels show a less alignment of sprout as compared to the upper region of the hydrogel (Turturro et al. 2013).

### Poly(lactide-*co*-glycolide) (PLGA)

A blend of polylactic acid (PLA) and polyglycolic acid (PGA) results in a copolymer called poly(lactide-*co*-glycolide) (PLGA). This copolymer has many important properties such as biocompatibility, mechanical strength, and its ease of manipulation in desirable shapes and sizes. This biodegradable polymer has been approved by FDA. The degradation rate and mechanical properties of this polymer are controllable and tunable due to which PLGA is extensively used in preparation of skin substitutes (Baoyong et al. 2010). The success of repairing process depends on the rate of epithelization which is synchronized with PLGA degradation rate resulting in completion of repairing process in a given time frame. A hybrid of natural polymer such as silk and PLGA electrospun scaffold has a combined effect on healing process of the wound (Chen et al. 2005).

### Polyethylene glycol (PEG)

Polyethylene glycol is a hydrophilic, biocompatible, non-immunogenic, flexible ether based polymer. These properties make it a desirable synthetic material for wound dressings. Growth factors such as EGF and PEG macromers have a good affinity and can be bonded together. These can be targeted to the wound site (Shahverdi et al. 2014). Properties such as mechanical, thermal and crystallinity of PEG are stabilized through blending the polymer with chitosan and PLGA. Diabetic wounds have been treated using these PEG based dressings. Such dressings help in wound healing through the initiating skin cells growth and their proliferation along with the deposition of collagen. Scar formation is also reduced by such dressing (Jeon et al. 2007).

### Polyurethane (PU)

Carbamate linkages join the organic units of polyurethane polymer. Polyurethane is used in wound dressing fabrication and it acts as a semi-permeable membrane protecting the wound from its surrounding environment and bacterial entry. Its semi-permeability provides a moist environment which is desired at the wound site. Adherence is a limitation of PU polymer which can be overcome by coating collagen or collagen-based peptides on polyurethane meshwork which greatly increase adhesion of the cells and also enhance biocompatibility of the tissue (El-Sayed et al. 2011).

## Polycaprolactone (PCL)

Polycaprolactone is obtained from degraded linear aliphatic polyester and autocatalyzed bulk hydrolysis. It possesses unique mechanical properties which make it a perfect biocompatible, biodegradable and bioresorbable polymer. It is non-toxic, FDA approved and can be easily processed into different shapes and forms (Jung et al. 2016). Electrospun PCL fibers are suitable to treat acute and chronic wounds. They mimic the fibrous structure of ECM. PCL material is limited by its poor antimicrobial properties. Therefore, silver nanoparticles are incorporated into PCL matrix to ensure its resistance to microbial invasion. In addition, the template uptakes sufficient wound exudate and has water retention capacities (Kakkar et al. 2014). PCL-collagen matrix acts as an excellent template that triggers integrin-β1 signaling pathway to regulate the growth of fibroblasts and initiates wound healing (Figueira et al. 2016).

## Natural polymer films as wound dressing

### Alginate dressings

Alginate dressings are mainly used to treat exudating wounds including infected post-operative wounds and leg ulcers. They are available in the form of porous sheets formed by freeze drying or fibrous materials intended for packing into wound cavities. When an alginate dressing comes in contact with an exuding wound, an ion exchange reaction takes place. Calcium ions in the alginate dressing are exchanged with sodium ions in the tissue fluid or wound exudates and the dressing swells. The degree of swelling of the dressing depends upon the chemical composition of the dressing and also on its botanical origin (Thomas 2000). Alginate dressings generally have high mannuronic or guluronic group contents.

One main reason for using alginates in wound dressings is their haemostatic ability. The coagulation effects of zinc and calcium alginate dressings have been compared with non-alginate dressings. It was found that alginate dressings were more effective as compared to non-alginate dressings in this respect. Zinc containing alginates had the best haemostatic ability (Segal et al. 1998).

Alginate dressings tend to remain gelled for as long a month, as compared to hydrocolloids which degrade sooner. In an experimental study, it was found that the lack of calcium concentration in the wound helped ‘gelling’ of the alginate dressing, while the presence of a specific concentration of calcium ions prolonged the degradation time of the gel (Ichioka et al. 1998).

Alginate dressings have applications in tissue regeneration and bio-engineering fields. Depending on composition, sodium alginate has been identified as a substrate for cell proliferation. This opens up new possibilities for tissue regeneration in skin scaffolds as well (Wang et al. 2003).

In another study on fetal rat chondrocytes cultured on alginate beads, the chondrocytes retained their original spherical shape and did not undergo de-differentiation. This supports the fact that alginate is a suitable medium for cell proliferation (Loty et al. 1998).

Wounds such as large leg ulcers and pyoderma gangrenosum exhibits low to high wound exudates. Dressings required for such wounds not only should have high absorptive capabilities but should also prevent the surrounding healthy skin from exudating wounds. These dressings must absorb the extra fluid while keeping the appropriate level of moisture for effective wound healing (Dabiri et al. 2016).

Alginate is a natural polymer that is extracted from brown algae by treating it with NaOH for commercial purposes (Clark and Green 1936). To form a precipitate of algae extracted it is further treated with NaCl or CaCl_2_. Alginic acid is produced by treating the precipitate with dilute hydrochloric acid. Alginate produced from bacteria may have better physical properties and chemical properties as compared to alginate that is extracted from seaweed (Rinaudo 2008).

Latest advancements in technology have led us to the development of nanoscale fibers that are used in wound management system. These nanofibers have many advantages including light weight, nanoscale fiber diameter, high surface area to volume ratio and porosity. Technique of electrospinning is used to produce alginate nanofibers. To improve the process, various synthetic polymers and/or surfactants are also used during the process (Rinaudo 2014).

When an alginate dressing comes in contact with an exuding wound, an ion exchange reaction takes place. Calcium ions in the alginate dressing are exchanged with sodium ions in the tissue fluid or wound exudates and the dressing swells (Boateng et al. 2008).The moisture level of the wound is kept in control by this gel formed. Alginate gels are able to absorb fluid about 20 times of their weight. This property of alginate gels makes them very desirable as dressing for wounds that contain low to high exudates (Dabiri et al. 2016). Modern wound dressings such as alginate dressing allow wound healing in moist environment (Orive et al. 2002).

Alginate being a natural polymer is biocompatible by confirmation through in vivo and ex vivo experiments. However, various impurities may still remain present in alginate as it is obtained from natural sources. These impurities can be of various types such as endotoxins, heavy metals, proteins and phenolic compounds that can cause immunogenic reactions at the site of implantation or injection (Orive et al. 2002).

However, in one of the experiments alginate highly purified by multistep extraction procedures and implanted into animals did not cause any immunogenic reactions. In another study, an alginate gel injected subcutaneously in mouse also did not cause any inflammatory reaction (Lee and Lee 2009).

Calcium alginate is regarded as a natural hemostat; therefore, alginate dressings can be used for wounds that are bleeding. Alginate dressings have an important property of gel formation which helps in reducing the pain during removal and changing of the bandage from the wound site. The rate of re-epithelialization and granulation is significantly increased which is provided by moist environment. In a controlled clinical experiment performed, calcium alginate proved to be more effective than the paraffin gauze dressings. When compared with the patients dressed with paraffin gauze, patients dressed with alginate gauze were completely healed at day 10.

Healing of split skin graft donor site is seen to be significantly improved by calcium alginate dressings. Bio-occlusive membrane dressings when used alone for treatment of split-thickness skin graft donor site cause the problems of pain and leakage along with seroma formation. Calcium alginate in combination with bio-occlusive dressings can be used to address these problems significantly (Paul and Sharma 2004).

Alginate gels can be also used for the delivery of drugs having low molecular weight. Small drugs are rapidly diffused through alginate gels which are commonly non-porous. The partially oxidized alginate gels are used for controlled and localized delivery of antineoplastic agents (Paul and Sharma 2004).

### Fucoidan

Fucoidan is a sulphated polysaccharide. It has gained a lot of interest in biomedical industry due to its anticoagulant property which is similar to that of heparin. Apart from that it also possesses other important properties such as anti-inflammatory, anti-viral, anti-thrombic and anti-tumoral effects. In recent years, fucoidan has found its use in wound and burn management due to its above mentioned properties (Murakami et al. 2010).

### Silk sericin

Silk sericin is a derivative of *Bombyx mori* and is a biocompatible protein. It contains amino groups along with hydroxyl and carboxyl groups in its composition. It has its use in wound dressings as it is known to have a positive effect on cell density and collagen formation at wound site by enhancing fibroblast growth and proliferation of human skin (Song et al. 2012). It has many advantages such as biocompatibility, non-toxicity and biodegradability. Due to these properties success full studies have been carried out on silk sericin for wound healing in biomedical industry. Wound of second degree burn can be covered using recombinant spider silk protein as a dressing material (Akturk et al. 2011).

### *Acetobacter xylinum*

*Acetobacter xylinum* utilizes carbon from nutrition media and form beta 1–4 glucose in the form of linear chains. These linear chains are secreted outside the cells where they assemble in a hierarchy from sub-fibrils through microfibrils to bundles of microfibrils. Subsequent formation of 3-D structure of ultrafine network and uniaxial orientation is enriched by cellulose (Evans et al. 2003).

Unique morphology of MC fibrils enables them to hold large quantity of water with nano-pores permitting transfer of medicine and blocking interaction with infectious agents, thus providing an effective physical barrier. Moreover, being free of lignin and hemicellulose it is set for a better non-pyrogenic, non-toxic and highly biocompatible biomaterial (Shah and Brown 2005).

### Keratin

Keratin is a naturally occurring biopolymer found in hair, nails, horns, wool feather and in epithelia of vertebrates. Its structure has a 3D mesh-like appearance which can take the form of hydrogel (Uppal et al. 2011). Keratin aids in healing process by interacting with the polyelectrolyte wound environment. Keratin can retain a large amount of water in its structure, which makes it a good candidate for wound dressing material as it can help in prevention of loss of biological fluids to the environment and reduce wound exudate. Its soft and moist nature along with its biocompatibility can be exploited to make it a promising dressing material for wound and burn treatment (Basu et al. 2017).

## Bioactive dressings made from natural polymers

Bioactive dressings are classified as those that play an active role in wound healing. Examples of bioactive materials that form part of these dressings are collagen, chitosan, hyaluronic acid and pectin (Dyson et al. 1988).

Collagen is secreted by fibroblasts in the body and is a common structural protein which stimulates cellular migration (Pereira and Bártolo 2015). Collagen has chemotactic properties that attract fibroblasts and thus enhances the deposition of new tissue during wound healing (Fleck and Simman 2010).

Gel dressings based on collagen are observed with reference to wound healing in genetically disabled mice. Collagen gel is applied to the wound fascia, covered and secured with Tegaderm. Granulation tissue formed on the collagen side of the dressing, along with wound contraction because of epithelial cell migration from surrounding tissue. Collagen promotes a greater degree of wound contraction, granulation tissue formation and angiogenesis. These characteristics make collagen ideal for open, dry wounds (Arnold-Long et al. 2010).

Antibacterial agents such as ciprofloxacin incorporated with collagen bi-layer dressings prevent the proliferation of invading bacteria in wounds that are susceptible to infection. The addition of an antibiotic to the dressing does not interfere with the ability of collagen to enhance granulation tissue formation and mediates successful closure of wound during the wound-healing process (Sripriya et al. 2007).

**Fig. 3:**
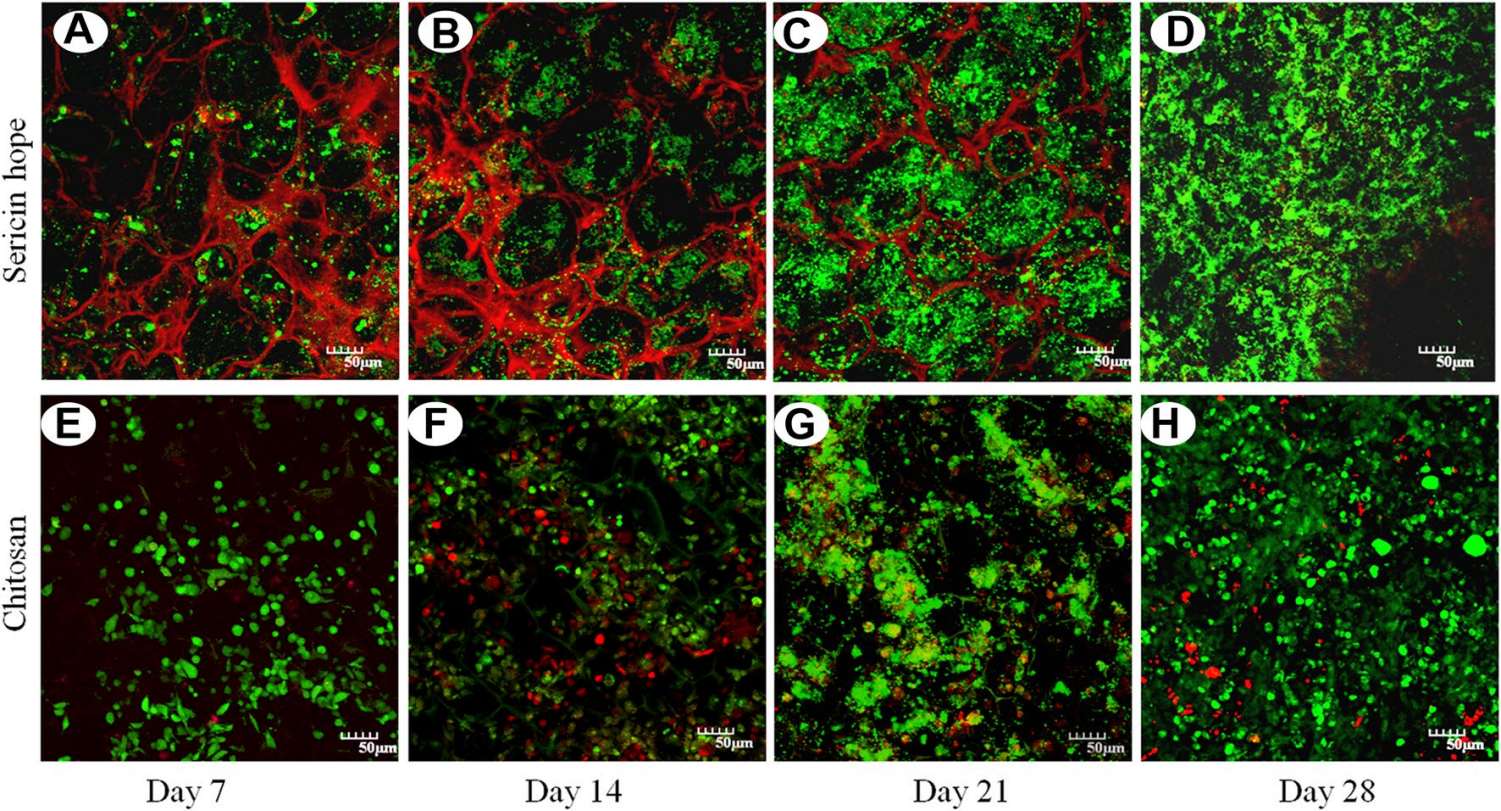
Cells cultured on chitosan and sericin porous matrices are shown using confocal microscopy. Dead cells produce red fluorescence and live cells produce green fluorescence (Nayak et al. 2013)

Biocompatible natural carbohydrate polymeric dressings including chitosan have been studied with respect to hemostasis in femoral wounds. Animal experiments done on 14 mixed breed swine have indicated superior hemostasis ability of chitosan as compared to gauze dressings. It has been hypothesized that chitosan dressings may be suitable for treating hemorrhagic wounds in prehospital settings (Gustafson et al. 2007). Chitosan is now categorized as a biomaterial that has biodegradable properties (Dai et al. 2011). It is known to accelerate wound healing by enhancing the activity of polymorphonuclear leukocytes (PML). Hence the organization and granulation of new tissue is augmented (Ueno et al. 2001). PEG enforced chitosan has been studied previously and the composite nature of the dressing has been shown to improve its mechanical properties. Composite biomaterials have the ability attain characteristics that may not be possible through the use of a single component (Petri 2015; Munib et al. 2015). In this case, chitosan inhibited inflammatory cell infiltration and promoted fibroblast activity, while PEG enhanced the migration of epithelial cells. The results of this study proved that such a dressing was effective in promoting wound healing (Chen et al. 2013). Viability of co-cultured cells in chitosan has been shown in Fig. 3.

In this figure, Alamar blue assay has been done to evaluate the cell proliferation on the sericin matrices at different time intervals. The sericin matrices show high proliferative response and low cytotoxicity up to 28 days. After 2 weeks, Alamar blue reduction in sericin and chitosan matrices were increased by 62 and 71%, respectively. On 3rd and 4th week, Alamar reduction for sericin matrices was 75–78% and for chitosan matrices reducation was 82–83%. Live/dead staining of culture matrices with fibroblasts and keratinocytes were performed at 7, 14, 21, 28 days to evaluate the cell viability. The existence of green and few red color shows the cell viability for 28 days.

Hyaluronic acid dressings have been used for temporary coverage of large skin defects until autologous skin grafts can be applied. In a case study on a tumor resection patient with a large forehead skin defect after surgery, hyaluronic acid dressing promoted good vascularization and granulation tissue formation. The wound indicated no hypertrophy or scarring after healing (Vindigni et al. 2011). Hyaluronic acid dressings have also been successfully employed for the treatment of skin lesions in pediatric purpura patients (Carella et al. 2013).

Chitosan coupled with hyaluronic acid have been used for the development of a dressing for skin ulcers. The dressings prepared were put through different hydration parameters which lead to the investigation of the mechanical properties of the dressing. Hydration tests were employed to test the ability of the composite dressing to absorb wound exudates. The addition of hyaluronic acid dressings decreased the hydration properties of the dressing and lead to a modulation of drug release (Rossi et al. 2007).

Pectin is another natural macromolecule that is part of many commercially available dressings and it is used along with cellulose and synthetic polymers. The acidic environment provided by pectin may act as a barrier for the growth of bacteria. ‘Duoderm’ and ‘Combiderm’ are some examples (Munarin et al. 2012). Alginate/pectin hydrogels for wound dressings are suitable as drug delivery agents as well, presenting a prolonged drug release profile which is dependent on the drug/polymer ratio (De Cicco et al. 2014).

Advances in the field of biomaterials have revolutionized medicine industry. Potential of microbes to produce various polysaccharides used for development of medical applications is a great leap ahead. Microbe-derived polysaccharides with innate physical and chemical properties have been investigated and exploited as potential biomaterial. Microbial cellulose (MC), a biopolymer, is produced by *A. xylinum* (Brown et al. 1976). MC’s physical and mechanical properties determine its range of application, from audio membranes to medical equipment.

During a span of past few years’ new avenues have been explored and developed to overcome discrepancies in the field of skin grafting and wound healing (Mir et al. 2014a, b). Various polymers are available in the market, which serve as a substitute for skin graft and wound dressings but quest for a polymer with properties and functions similar to human skin, is continued. Being similar to plant’s cellulose (MC) serves a perfect matrix for wound healing (Rojas 2016).

Wound healing is a complex process which involves myriad interactions between various cells. Research on wound healing encompasses identification of elements that accelerate healing process and reduce scaring, materials that serve as effective skin substitutes and signals responsible for triggering healing rather than scaring. Such wound-healing system should be devised that provides milieu for optimal epidermal regeneration and a barrier against wound infection and fluid loss (Quinn et al. 1985; Berthet et al. 2016).

Use of natural polymers is prevalent in medicine, for wound and burn dressings, because of biocompatibility, biodegradability and similarity to ECM. Varying greatly in their chemical properties, polysaccharides are used in wound and burn management. Polysaccharides used for this purpose can be acidic, basic or sulfated.

### Homoglycans

Biochemically, homoglycans are made up of monomers which are repeated throughout the chain length. Homoglycans occur naturally in the form of starch, cellulose, and dextran (Lee et al. 2009). Being a biocompatible material has enabled them to act as a modulator for cellular responses involved in wound healing (Lloyd et al. 1998).

### Pallulan

Pallulan, biosynthesized from starch, is used as wound dressing. Pallulan derivative is used to make superabsorbent hydrogel lints which can be loaded with antibacterial and anti-mycotic drugs. Controlled release of these drugs prevents formation of biofilm thus protecting injured and burned areas (Li et al. 2011a, b).

### Yeast

Yeast, grains and fungi yield beta glucans which can form double and triple helix resistant gel. This natural polymer supports the process of wound healing and reduces skin irritation. Dextran derivatives, being of natural origin, are biocompatible and used as wound dressings. They control the proliferation of cells in a biofilm. Their conformation render them odor controlling dressings (Logeart Avramoglou and Jozefonvicz 1999).

### Cellulose and chitin

Cellulose being a natural polymer, exhibits great potential as wound dressings because it stimulates the process of granulation and epithelialization (Medusheva et al. 2007). Chitin is a natural amino polysaccharide present invertebrates, crustacean and insect exoskeleton as cell wall of fungi. Annual production of chitin is at par with cellulose. Chitin is biocompatible and bioactive. When co-cultured with keratinocytes and fibroblasts, they serve as films, hydrogels and sponges for wounds and burns (Kucharska et al. 2002; Dai et al. 2011).

### Alginates

Alginates, obtained from processed algae, are unbranched polysaccharides used as wound dressings. Alginates are absorbent in nature and form hydrophilic gels, providing a moist wound-healing environment (Qin et al. 2006; Thu et al. 2012).

### Agar

Natural agar and agarose polysaccharides are used in dressing applications. These natural fibers have water absorbing capacity thus making them an ideal candidate for wound dressing (Bao et al. 2010).

### Pectin

Pectin is biocompatible and biodegradable polymer with a wide range of applications in food industry (Berthet et al. 2016). It is also used as moist dressing for wound and burn management (Mishra et al. 2012).

### Bovine serum

Nanofibers of bovine serum albumin are prepared from the globular protein through electrospinning. Bovine serum albumin being biocompatible, biodegradable, and good mechanical properties may be used in biomedical applications such as for suturing and dressing for wounds and burns (Dror et al. 2008; Shah and Brown 2005).

Management of wounds and burns is important for prevention of site specific infection. Wound dressing plays an important role. Various natural polymers with biocompatibility and stability are used in medicine for wound and burn dressing thus eliminating perils of persistence in the body (Das and Baker 2016).

### Engineered skin substitutes for wound healing

In spite of major developments in the field, there are currently no dressings that will completely simulate the functions of healthy, uninjured skin. Bio-engineering for skin substitutes has provided an option for the healing of recalcitrant chronic wounds have failed to heal by the use of ordinary dressings. The following types of tissue engineering techniques and skin substitutes have been discussed:

### Bi-layered bioengineered and self-assembly skin substitutes

Bi-layered bioengineered skin substitute (BBSS) also known as ‘apligraph’ has been used for the treatment of diabetic foot ulcers and venous leg ulcers. The skin substitute is composed of both epidermal and dermal layers formed from keratinocytes and fibroblasts respectively. The product has been tested in clinical trials with no incidence of clinical rejection. BBSS may be used as an adjuvant to conventional therapy for venous leg ulcer patients (Curran and Plosker 2002). Skin substitutes made by the ‘self-assembly approach’ have been employed successfully in the treatment of chronic ulcers. This approach has been used in combination with compression therapy, and might hold promise as an adjuvant therapy for patients who are unresponsive to traditional therapy alone (Boa et al. 2013).

### Bioengineered dermal preparations

Cultured allograft and autograft epidermal sheets are known for enhancing wound healing in burn wounds. Nonetheless, these constructs lack a dermal layer, which may help in granting good mechanical strength to the wound as well as aiding wound contraction. Allografts from cadaveric skins are another option, however, the time period for their use is limited by the problem of host rejection. To counter this, preparations are in use that have had the immunogenic components removed. ‘Alloderm’ (Life Cell Corporation) is one such example. Such grafts may be used in combination with autologous keratinocytes for improved results (Cuono et al. 1986a, b). Acellular (collagen) matrixes from porcine small intestines (Oasis, Cook Biotech Inc.,) are shown to have a long shelf life. A clinical trial done on 120 venous leg ulcer patients has exhibited enhanced healing after a period of 84 days. This clinical trial was a comparison between patients receiving compression therapy alone, with patients treated with the skin substitute, along with compression therapy(Mostow et al. 2005).

### Tissue-engineered skin substitutes

**Fig. 4:**
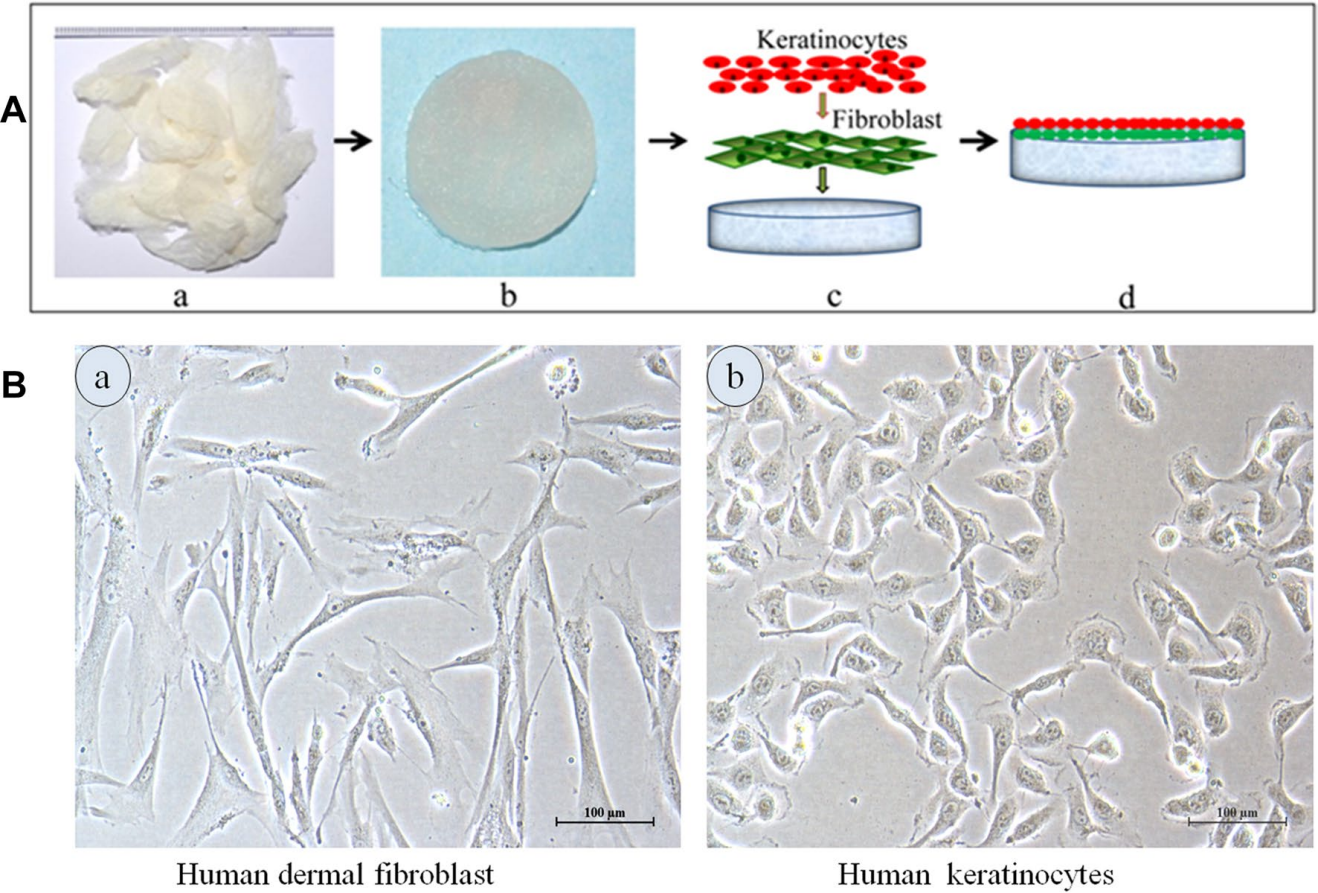
Co-culture of cells in fibroblasts and keratinocytes has been seeded on sericin matrices to create a tissue-engineered skin substitute. **a** Fibroblasts and **b** keratinocytes (Nayak et al. 2013)

The adherence and interaction of cells depend upon integrin proteins that interact with the extracellular matrix. Such mechanisms ultimately influence downstream signaling pathways of cells, and thus the organization of development of new tissue. The most common biomaterials used in tissue engineering scaffolds for wound healing includes collagen. Collagen is laid down by fibroblasts in the skin, and is arranged in the form of fibrils such that its structural capacity allows the tissue to bear shear and tensile forces without rupture or damage. Collagen has been used in the production of an endothelialized matrix that promotes the formation of a capillary like network of blood vessels (Hudon et al. 2003). Collagen–glucosamine skin scaffolds have been shown to regenerate epidermis and dermis in vivo and also promote vascularization in these structures, in porcine models (Cuono et al. 1986a, b). Figure 4 depicts fibroblast cell proliferation on sericin (silkworm) matrices.

Conditions in which normal auto grafts cannot be used to replace the damaged skin often lead to the death. There is a significant need for the tissue-engineered skin substitutes (Shevchenko et al. 2009). Substantial progress has been made in the development and clinical use of bioengineered skin substitutes (Thomas 1990). Tissue-engineered skin substitute in the field of wound healing is very significant. Tissue-engineered skin substitutes classified either anatomically (epidermal, dermal, or composite), type of biomaterial (xenograft, allograft, or autologous graft), cellular component (cellular and acellular), or depend upon the temporary, semi-permanent or permanent) (Rheinwatd and Green 1975).

### Cultured epidermal autografts (Epicel™)

Rheinwald and Green developed a technique in 1975 that epidermal keratinocytes to be cultured in vitro using a feeder layer of irradiated murine fibroblasts (Green et al. 1979). Dermis and subcutaneous tissue are removed from the skin. Epidermis is crushed and trypsinized, and a cell suspension is made on lethally irradiated 3T3 mouse fibroblast. After cell suspension is plated, small colonies are produced within a few days. After that when a number of colonies reaches at maximum level, the keratinocytes are released and adhere to the non-adherent gauze dressing (Hefton et al. 1986). Two to three weeks are required to produce grafts after the biopsy is obtained. Cultured epidermal autografts (Epicel™) were first produced in 1988 by using this technique. The advantage of this autografts is to provide a permanent wound covering (Gallico et al. 1984), faster pain relief and decreased the requirement of the donor sites. The disadvantage of this technique is a 3 week delay for the graft cultivation and the lack of dermal components. The high cost is also the major factor. The cultured epidermal autografts have been used to treat burns (Romagnoli et al. 1990), chronic leg ulcers (Gallico et al. 1984), congenital hypospadias (Carter et al. 1987), epidermolysis bullosa (Phillips and Pachas 1994), pressure ulcers (Pellegrini et al. 1997) and corneal replacement (Heck et al. 1985). A cadaveric dermal allograft combined with the cultured autologous epidermal was used in treatment of 8 patients with burns (Cuono et al. 1986a, b). This cadaver allograft left for 4 days, after that autologous epidermis was grafted to the exposed dermal surface (Eisenbud et al. 2004). However, this is not considered as the ideal treatment for the permanent skin replacement. Only partial success was achieved (Phillips 1993).

### Cultured epidermal allografts

Cultured epidermal allografts are derived from the allogeneic donors such as newborn foreskin. They promote the formation of granulation and stimulate the epithelialization in the edges of wound and adnexal structures in the dermal bed of shallow wound (Leigh et al. 1987). The advantage of this technique is to produce immediate graft. The disadvantage of these allografts is that they do not permanently survive on wound area and maybe there are the chances of disease transmission. This disease transmission can be reduced with extensive screening. These cultured epidermal allografts are used to treat chronic leg ulcers, (Phillips et al. 1989, 1993; Duhra et al. 1992; Hefton et al. 1983), burns (Madden et al. 1986; De Luca et al. 1989), donor sites (Mcguire et al. 1987) and epidermolysis bullosa (Mcguire et al. 1987).

### Dermal substitute

Dermal substitutes are engineered using the freeze drying, sterilization and decellularization (Mcguire et al. 1987). The main purpose of the dermal scaffolds are to act as temporary or permanent wound covering to enhance the healing. They have been used in burns, ulcers and surgical wounds (Debels et al. 2015; Shahrokhi et al. 2014).

### Acellular xenografts

Animal derived acellular xenografts having the long shelf life and do not transfer any infectious material. EZ-Derm^®^ (Molnlycke Health Care AB) is a collagen matrix derived from the procine dermis that is crosslinked and filled with silver to prevent any rejection or infection (Clark et al. 2007). Biobrane™ (UDL Laboratories) is a nylon mesh, crosslinked with the porcine collagen. This is a bi-layered substitute with a removable semi-permeable silicone layer that acts as temporary epidermis. In comparison to the Duoderm, Biobrane is failed to heal the wound of intermediate thickness burn and skin graft donor sites. The disadvantage of Biobrane is to attach tightly and damage the new granulation tissue (Heimbach et al. 1988). Burke and Yannas developed the artificial skin, Integra^®^. It is made up of a shark derived matrix of bovine collagenase chondroitin-6-sulfate and disposable silicone sheet. The disposable epidermis is removed and it is replaced with the split-thickness skin graft when matrix becomes vascularized. The FDA approved it for the treatment of burns in 1996. The advantage of this product is to develop the neo-dermis and the disadvantage is that unseen infection can occur under the Integra^®^. Studies showed Integra having the greater foreign body reactivity and less wound contraction as compared to AlloDerm^®^ (Yang et al. 1989). Integra showed greater foreign body reactivity and less wound contraction compared to human-derived dermal collagen matrix AlloDerm^®^ (Yang et al. 1989). In a controlled, randomized, multicenter study of Integra^®^ in 106 burn patients, the median artificial dermis ‘take’ was 80% compared with 95% for all comparative sites, but this ‘take’ was equivalent to that of all non-autograft control materials (allograft, xenograft or synthetic dressing). Donor sites used for the study site were significantly thinner and healed faster than control sites (Truong et al. 2005). An alternative therapy has been reported in three burn patients, using a combination of Integra^®^ plus skin biopsies to prepare cultured skin substitutes, followed 3 or more weeks later by removal of the silastic cover of Integra^®^ and application of the cultured skin substitutes (Vanstraelen 1992).

OASIS wound matrix is derived from porcine small intestine submucosa and contains the collagen, hyaluronic acid, proteoglycans, fibronectin and growth factors such as FGF-2 and TGF- β (147). It is approved for the diabetic, venous and pressure ulcers, second degree burn, graft donor sites but not approved for the surgical wounds. In a multicenter study, OASIS compared with the Hyaloskin^®^ (Apeldoorn) in which 82% treated with OASIS healed completely as compared with the 46% of Hyaloskin-treated patients after 16 weeks. In a study comparing OASIS with a pure hyaluronic acid matrix Hyaloskin^®^ (Apeldoorn) in mixed venous–arterial ulcers, 82% treated with OASIS healed completely compared to only 46% of Hyaloskin-treated ulcers after 16 weeks. OASIS showed less pain compared to Hyaloskin (Evans et al. 2003). OASIS is promising for wound healing compared to other crosslinked products (Boyce et al. 1999).

### Acellular allografts

Acellular dermal allografts are made up from cadaveric skin and have been used to remove infectious agents and cellular materials. AlloDerm, KaroDerm, SureDerm and Graft Jacket are the acellular allografts (Rheinwatd and Green 1975).

AlloDerm^®^ has been used since 1992. Derma Matrix™ (Synthes, West Chester, PA) also in the same category of cadaveric-derived acellular dermal allografts. One study is conducted on mouse model in which multiple surgical wounds treated with different skin substitutes. AlloDerm grafts has become softer and poorly clear in 3 months after implantation while Derma Matrix maintained its reliability (Martin et al. 2002). Neox^®^ is derived from the human placenta. It is not crosslinked and contains the fibronectin, collagen and similar to dermal skin (Purdue et al. 1997).

Since 1992, a controlled and randomized clinical study on 67 burn patients in a controlled multicenter clinical study has shown that results have been the same for both thin split autografts with allograft dermal matrix and thick split autografts alone (Marston et al. 2003). Immunologically is inert and it has the advantage of dermal matrix. The disadvantage of dermal matrix is that it has the risk of transmitting the infectious material and requires two surgical procedures (Bello et al. 2001).

### Acellular synthetic dermal substitutes

Collagen, hyaluronic acid, fibronectin and alginates are the naturally occurring material that is present in the extracellular matrix of the human produced acellular synthetic dermal substitutes. Hyalomatrix^®^ (Anika Therapeutics) is composed of hyaluronic acid which is non-crosslinked with outer silicone membrane used in burns, diabetic ulcers, and chronic wounds (Still et al. 2003),

## Skin substitutes containing allogenic live fibroblasts

### TransCyte^®^

TransCyte^®^ is the first FDA approved temporary bioengineered skin substitute (Martin et al. 2002). It is made up of neonatal fibroblasts which are cultured on nylon fiber that are embedded into a silastic layer for 4–6 weeks. Dense cellular tissue is formed (Shevchenko et al. 2009). It is similar to the Biobrane. TransCyte showing faster wound healing as compared to the Biobrane in one study (Mcguire et al. 1987; Sibbald et al. 2005). A randomized and controlled study in 66 patients with 132 removed burn wounds showed that TransCyte is effective as cadaver skin (Hasegawa et al. 2004).

### Dermagraft^®^

Dermagraft^®^ contains the absorbable polyglactin scaffold colonized with allogenic neonatal fibroblasts. In controlled and randomized study of chronic diabetic ulcers, 30% patients treated with dermagraft were completely healed after 6 weeks as compared to the 18% patients which were healed after 12 weeks, treated with standard wound care (Lee 2000).

### Composite graft

Two commercialized products have been developed: Orcel (Forticell Bioscience, Inc, New York, NY, USA) and Apligraf (Organogenesis, Inc, Canton, MA, USA). These products are made up of collagen scaffold, cultured fibroblasts and a layer of stratified cultured human keratinocytes.

### OrCel

OrCel is an FDA approved for the treatment of epidermolysis bullosa and split-thickness skin graft donor sites (Supp and Boyce 2005; Límová 2010; Trent and Kirsner 1998). It is made up of cultured neonatal keratinocytes and bovine collagen. It is used for the treatment of venous ulcers (Bello and Phillips 2000).

### Apligraf

Apligraf an example of a “composite skin graft”, “skin equivalent” or “organo-typical skin substitute” as it is composed of both living dermis and epidermis. In 1998, FDA approved it for clinical use as first composite skin graft for the treatment of venous ulcers or neuropathic diabetic ulcers (Bello and Phillips 2000; Falanga and Sabolinski 1999). Apligraf is an only commercially available composite bi-layer product which is approved by FDA for the treatment of chronic venous ulcers (Veves et al. 2001; Zaulyanov and Kirsner 2007). Patients, treatment with apligraf, experienced faster healing and decreased the complication rate, by this it meant less need for medical follow up (Límová 2010; Supp 2011). Apligraf accelerates healing in diabetic neuropathic ulcers as compared with standard therapy of moist gauze and offloading (Supp 2011).

To obtain FDA approval, two successful trials were performed to treat VLU and DFU by using apligraf. For VLU, the title was “a randomized controlled trail of the allogeneic human skin equivalent was evaluated for the safety, efficacy and immunological impact in the treatment of venous ulcers” (Límová 2010). The experiment was performed on 293 patients and examined VLU. After 6 months, 63% treated patients treated with apligraf were completely healed as compared to the 49% patients treated with conventional therapy. This study found that bi-layer bioengineered skin was much better than the conventional therapy. Adverse effects were the same for both groups but there is no clinical rejection found in bioengineered skin treatment. This was the first study that proving apligraf is an effective and safe mode of treatment for chronic non-healing venous leg ulcers (Hong et al. 2006).

For DFU, title was “A randomized controlled prospective trial investigating the effectiveness of apligraf in the treatment of non-infected, non-ischemic, chronic plantar diabetic foot ulcers” (Vindigni et al. 2011). The experiment was conducted on 208 patients in 24 different centers of US. After 12 weeks, 56% patients treated with apligraf attained complete wound healing in comparison with 38% of the control group. Adverse reactions were similar in both groups. Osteomyelitis and lower limb amputations were less common in the group treated with living skin graft. This study showed the benefits of apligraf (Hong et al. 2006).

In the last few decades, there has been a remarkable increase in the development of bioengineered skin substitutes. A wide array of biologically active materials and skin substitutes has been developed. Some advance membranes that contain the live cultured cells, allogeneic or autologous; attempt to regenerate skin using the fibroblasts and keratinocytes in a scaffold that can apply into wound that leads to its healing (O’Leary et al. 2002).

Chemically crosslinked products are less appropriate for skin substitutes. Biocompatibility can be enhanced by mixing these products with fibroblasts to allow natural remodeling process before application. Research is needed to reach the potential of fibroblast seeded scaffolds while the high production costs can form a serious issue (Alrubaiy and Al-Rubaiy 2009). The treatment of malignant wounds with skin substitute has not been reported but this possibility should be kept in mind (Romanelli et al. 2007).

Adipose-derived human lipoaspirate from embryonic mesenchyme contains growth factors that promote healing (Zaulyanov and Kirsner 2007). Although stem-cell research is still at its beginning, there are rapid ongoing developments.

The future looks to be promising for skin substitutes. Artificial skin may be very helpful in many aspects. Some studies have shown that skin equivalent keratinocytes are in an activated state (Shevchenko et al. 2010). This increases the theoretical possibility that such cells may have an increased risk of future malignancies or perhaps some physiological differences during wound healing or skin aging. In future, one development may be tried to reiterate more of the properties of in vivo skin (Shevchenko et al. 2010).

Living cell therapy looks to be most promising field of wound healing. It has the advantage for the physician to treat the variety of wounds (Shevchenko et al. 2010).

### Non-contact radiant heat bandages

Non-contact radiant heat bandages have also been tested as a potential aid in wound healing. A non-contact radiant heat bandage consists of a power supply and a heating element, attached to two flaps of polymeric film. A window for visualization of the wound is also present, so that wound condition can be analyzed once the heating element is removed. Studies have been conducted using Ovine Staphylococcus Aureus dermal infections as the experimental model. The use of local radiant heat had the effect of controlling the spread of infection and promoting wound healing (Lee et al. 2000). This might be explained by the fact that the application of local heat to a wound has been known to increase oxygen tension, thus increasing resistance to infection. Randomized trials involving local radiant heat therapies have shown faster healing rates in patients with pressure ulcers as compared to the control (Ikeda et al. 1998; Thomas et al. 2005).

## Conclusion

A wound dressing thus approaching ideal characteristics should conform to the site of the wound, offer alleviation of pain symptoms, promote faster wound-healing time and attempt to restore the patients’ normal daily activities. This review of literature highlights the need for a more holistic approach towards wound healing and management so that while selecting the appropriate dressing for a wound, the physiological and biochemical requirement of the wound and the patient are also taken into account.
